# A New Method Combining Finite Element Analysis and Digital Image Correlation to Assess Macroscopic Mechanical Properties of Dentin

**DOI:** 10.3390/ma8020535

**Published:** 2015-02-06

**Authors:** Wenlong Wang, Nicolas Roubier, Guillaume Puel, Jean-Marc Allain, Ingrid C. Infante, Jean-Pierre. Attal, Elsa. Vennat

**Affiliations:** 1 MSSMat, UMR 8579 CNRS-Ecole Centrale Paris, Grande Voie des Vignes, 92295 Chatenay-Malabry Cedex, France; E-Mails: wangw@ecp.fr (W.W.); nicolas.roubier@ecp.fr (N.R.); guillaume.puel@ecp.fr (G.P.); 2 LMS, UMR 7649 CNRS-Ecole Polytechnique, 91128 Palaiseau Cedex, France; E-Mail: allain@lms.polytechnique.fr; 3 SPMS, UMR 8580 CNRS-Ecole Centrale Paris, Grande Voie des Vignes, 92295 Chatenay-Malabry Cedex, France; E-Mail: ingrid.canero-infante@ecp.fr; 4 URB2i, EA4462 Université Paris Descartes, 1 rue M. Arnoux, 92120 Montrouge, France; E-Mail: jean-pierre.attal@parisdescartes.fr

**Keywords:** dentin, three-point bending test, digital image correlation, finite element analysis, mechanical properties, stress and strain assessment

## Abstract

A literature review points out a large discrepancy in the results of the mechanical tests on dentin that can be explained by stress and strain assessment during the tests. Errors in these assessments during mechanical tests can lead to inaccurate estimation of the mechanical properties of the tested material. On top of that, using the beam theory to analyze the bending test for thick specimens will increase these experimental errors. After summarizing the results of mechanical tests on dentin in the literature, we focus on bending tests and compare the stress assessment obtained by finite element analysis (FEA) and by beam theory application. We show that the difference between the two methods can be quite large in some cases, leading us to prefer the use of FEA to assess stresses. We then propose a new method based on coupling finite element analysis and digital image correlation (DIC) to more accurately evaluate stress distributions, strain distributions and elastic modulus in the case of a three-point bending test. To illustrate and prove the feasibility of the method, it is applied on a dentinal sample so that mean elastic modulus and maximum tensile stress are obtained (11.9 GPa and 143.9 MPa). Note that the main purpose of this study is to focus on the method itself, and not to provide new mechanical values for dentin. When used in standard mechanical testing of dentin, this kind of method should help to narrow the range of obtained mechanical properties values.

## 1. Introduction

Dentin is the main mineralized biological tissue of the tooth. It is located between the enamel and the pulp cavity and shows a hierarchical and complex structure at different scales. At the nanoscale, it consists of a carbonated nanocrystalline apatite mineral phase (approximately 50% by volume), a grid work of type I collagen fibrils (approximately 30% by volume) and fluids (approximately 20% by volume) [1]. At the microscale, dentin can be seen as a continuous fiber-reinforced composite with peritubular cuffs as reinforcement and a matrix of intertubular dentin. At the macroscale, it can be seen as a bulk material with effective properties resulting from its complex structure. The knowledge of these properties is crucial to predict the tooth’s response to applied loads [2]. Dentin mechanical properties assessment is also important in order to improve its restoration and to realize more biomimetic restorative materials.

**Figure 1.**
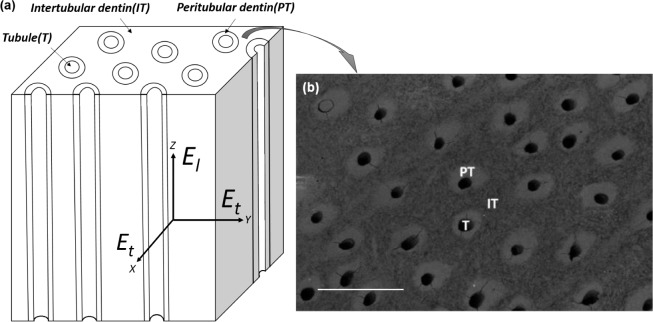
(**a**) Dentinal structure: the directions of longitudinal modulus (*E_l_*) and transverse modulus (*E_t_*) are shown in the scheme. (**b**) Scanning electron microscopy image from the top view of dentin, with the identification of regions of peritubular dentin (PT), intertubular dentin (IT), and a tubule (T). Scale bar corresponds to 10 *μ*m.

The specific microstructure of dentin described above obviously influences the dentinal mechanical properties. Considering dentin as a composite made of tubules, peritubular and intertubular dentin, as shown in Figure 1, dentin can be approximately modeled as a transverse isotropic material [2,3].

Thus, the relation between stress ***σ*** and strain *ε* in linear elasticity could be described by the classical relationship [4]:

$$\left[\begin{array}{c} \varepsilon_{xx}\\ \varepsilon_{yy}\\ \varepsilon_{zz}\\ 2\varepsilon_{yz}\\ 2\varepsilon_{xz}\\ 2\varepsilon_{xy} \end{array}\right]=\left[\begin{array}{cccccc} \frac{1}{E_{t}} & -\frac{v_{tt}}{E_{t}} & -\frac{v_{tl}}{E_{t}} & 0 & 0 & 0\\ -\frac{v_{tt}}{E_{t}} & \frac{1}{E_{t}} & -\frac{v_{tl}}{E_{t}} & 0 & 0 & 0\\ -\frac{v_{tl}}{E_{t}} & -\frac{v_{tl}}{E_{t}} & \frac{1}{E_{t}} & 0 & 0 & 0\\ 0 & 0 & 0 & \frac{1}{G_{tl}} & 0 & 0\\ 0 & 0 & 0 & 0 & \frac{1}{G_{tl}} & 0\\ 0 & 0 & 0 & 0 & 0 & \frac{2(1+v_{tt})}{E_{t}} \end{array}\right]\left[\begin{array}{c} \sigma_{xx}\\ \sigma_{yy}\\ \sigma_{zz}\\ \sigma_{yz}\\ \sigma_{xz}\\ \sigma_{xy} \end{array}\right]$$

where *E_t_*, *E_l_* are the transverse and longitudinal modulus respectively, *ν_tl_*, *ν_tt_* are the associated Poisson’s ratios, and *G_tl_* is the shear modulus in longitudinal direction.

These transverse and longitudinal moduli but also the maximal tensile and compressive stresses have been widely investigated experimentally since the middle of the last century [2], using either compression or traction experiments. Through tensile tests, the elastic modulus (*E_t_*) have been estimated by Sano *et al.* [5] to be 13–15 GPa. When using an optical strain gauge to determine the strain [6], the elastic modulus was reported to be 19.3 GPa but with a larger deviation (28%). Through compressive tests, the elastic modulus of human dentin was determined by Peyton *et al.* [7] to be 11.6 GPa. Strain gauges were attached to steel rods that were then used to apply the load to the specimens. A compressive modulus value of around 14 GPa can be found in the literature [8,9]. These lower moduli values obtained in compression may arise from the non-parallel alignment of load and possible relaxation effects. Craig and Peyton [10] reported a modulus of 18.5 GPa modified by load–unload cycles with the strain gauge directly onto the sample to remove the effect of the compression setup rigidity. Recently, Zaytsev *et al.* [11] reported elastic modulus values within a range of 3 to 13 GPa as a function of the geometry of the sample. However, they did not explicitly indicate the method used for the strain measurement, which made it difficult to place their results within the framework of others. Finally, diametral compressive and classical compressive tests were performed by Palamara *et al.* [8] using digital image correlation (DIC) to determine elastic modulus and the values were 6.5 *±* 2 GPa and 10.7 *±* 2.4 GPa respectively. In all these tests, from the distinct strain rates we deduce that the results are likely to be influenced by dentin viscoelasticity: Jantarat *et al.* [12] have already reported a linear dependence of elastic modulus as a function of strain rate.

Bending tests seem to be more rarely used, probably due to the fact that a special setup is needed to carry out such tests. Nevertheless, three-point [13] or four-point [14] bending tests can be used to assess dentin mechanical properties. Rees *et al.* [13] determined a static elastic modulus (of 8.6 GPa with a standard deviation of 0.86 GPa) using a three-point bending test and a dynamic modulus ranging from 14.3 to 15.9 GPa in the frequency range of 0.1 to 10 Hz. Ryou *et al.* [14] obtained an elastic modulus (13–17 GPa) depending on the location in dentin with four-point bending tests.

Some other techniques were also used to determine dentin mechanical properties. Transverse isotropic elastic constants along orthogonal directions to the tubules (*E_t_*) were indeed studied by Lees and Rollins [4] using critical reflectance ultrasound spectroscopy (RUS) measurements. A modulus of 36 GPa in the tubule direction (*E_l_*) and 29 GPa in the perpendicular axis (*E_t_*) was reported. Differing values (from 23 to 25 GPa) were reported by Kinney *et al.* [3] using resonant ultrasound spectroscopy measurements, but without assumption on material symmetry. More recently, anisotropic properties depending on the tubule orientation were investigated through micro-pillar compression tests [15–17], the moduli were reported from 3.5 to 16 GPa as a function of the tubule orientation. In this study, ten tubules per pillar can be observed, which might be slightly lower than what should be found in a Representative Volume Element (RVE) [18].

Table 1 summarizes the different values of dentin elastic modulus and ultimate strength found in the literature. We note that a wide range of values can be found and a great number of methods for stress and strain assessments have been used, indicated if available.

In order to narrow the range of dentin mechanical properties values, a new method to assess stress and strain in the case of a three-point bending test is proposed. A two-step procedure is thus chosen:

First, for typical, published, bending tests, we propose to re-evaluate the stresses by the finite element method instead of using the Bernoulli–Euler beam theory as it is classically done. The differences in terms of maximum stress will be illustrated through the analysis of two articles [13,19].

Secondly, a three-point bending test combined with Finite Element Analysis (FEA) and Digital Image Correlation (DIC) is proposed, taking advantage of the complementarity of these two methods.

The DIC technique is increasingly used to investigate the mechanical behavior of human tissues [20] in that it enables the obtainment of the whole strain field on a surface. However, this technique is not commonly used in dental material testing: to our best knowledge, only two studies on dental materials used DIC [8,20]. At the same time, FEA allows the most accurate evaluation of stresses in complex configurations but has not been used to assess stresses during dentin mechanical testing to the authors’ knowledge. Surprisingly, there have been few studies using this combination of two methods (FEA and DIC) to determine the elastic modulus of dentin.

## 2. Experimental Section

### 2.1. Recalculation of the Stresses in the Bending Test Configurations Found in the Literature

Two articles were chosen because they provide sample geometries and some results from three-point bending tests and four-point bending tests respectively [13,19]. The values from the published articles were recalculated using the classical beam theory (Sections 2.1.1 and 2.1.2). Then, using the experimental conditions mentioned in the two selected articles, the stresses were recalculated by the finite element method (Section 2.1.3).

#### 2.1.1. Three-Point Bending Test [13]

**Figure 2.**
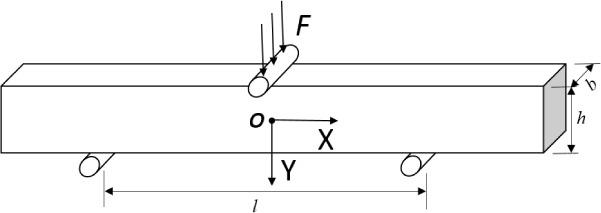
Scheme of three-point bending test.

Rees *et al.* [13] performed static three-point bending tests to measure dentin elastic modulus (Figure 2).

To assess the stresses in the beam, the Bernoulli–Euler beam model can be used (at *x* = 0, which is the cross-section where the stresses are the highest) with:

$$\sigma_{xx}=\frac{3Fl}{bh^{3}}y$$

where *σ_xx_* is the normal stress in direction *x*, *F* is the load, *l* is the supporting span, *b* is the beam width, *h* is the beam thickness, and *y* is the vertical coordinate.

In the Rees *et al.* experiment [13], beams were 2 mm wide, 2 mm in depth and presented an average length of 13 mm. The distance for the supporting span was 11 mm. Thus, the ratio (*l/h*) was 5.5. Then, the modulus was calculated from Bernoulli–Euler beam equation:

$$E=\frac{Fl^{3}}{4bdh^{3}}$$

where *F* is the load, and *d* is the deflection at the given load.

#### 2.1.2. Four-Point Bending Test [19]

**Figure 3.**
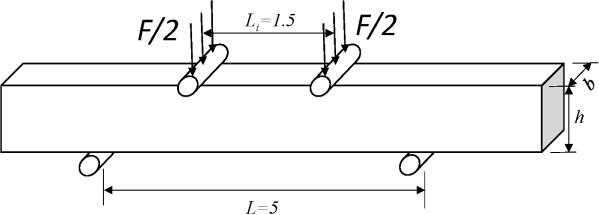
Scheme of four-point bending test.

Eltit *et al.* [19] performed four-point bending tests on dentinal samples. They studied the maximum flexural stress of coronal and root dentin. The tested beam dimension was 0.4 mm × 1.2 mm × 7 mm (thickness × width × length). The supporting span of the bending device was 5 mm, and the loading span was 1.5 mm, as is shown in Figure 3.

According to the Bernoulli–Euler beam equation, the maximum flexural stress can be expressed as:

$$\sigma_{f}=\frac{3F(L-L_{i})}{2bh^{2}}$$

where *σ_f_* is the maximum flexural stress, *F* is the load, *L* is the supporting span, *L_i_*, is the loading span, *b* is the beam width, and *h* is the thickness of the beam.

#### 2.1.3. Recalculations by Finite Element Analysis (FEA)

The simulations were performed with the commercial software COMSOL Multiphysics (Comsol Co., Stockholm, Sweden). In the FEA, we use the static equilibrium equations under small deformation assumption with an isotropic relation:

$$\begin{array}{c} f_{b}+\nabla \cdot \sigma =0\\ \varepsilon =\frac{1}{2}[\nabla u+\nabla u^{T}]\\ \varepsilon =\frac{1+v}{E}\sigma -\frac{v}{E}tr(\sigma )I \end{array}$$

where ***f****_b_* is the body force, ***σ*** is the stress tensor, *ε* is the strain tensor, *u* is the displacement, *ν* is the Poisson ratio, *E* is elastic modulus, and **I** is the identity matrix.

For recalculating the stress in three-point bending test, about 204,000 tetrahedral elements were used in the mesh. The boundary conditions are the following:

- Fixed constraints (*u* = 0) were applied to the two support edges.
- Boundary load of total force (50 N) was applied to the middle area (0.6 mm × 2 mm) between the two supporting edges.

For recalculating the stress in four-point bending test, about 240,000 tetrahedral elements were used in the mesh. The boundary conditions are the following:

- Fixed constraints (*u* = 0) were applied to the two support edges.
- Edge load of total force (5 or 12 N) was applied to the loading part (consisting of two loading edges here).

The edge load was chosen in order to reproduce data from the Eltit *et al.* [19] experiment. Within the load-deflection curves from the four-point bending tests of Eltit *et al.* [19], it can be inferred that the maximum bending force used for crown dentin beams was about 5 N, and for root dentin beams, the bending force was around 10–15 N (here, 12 N was chosen as an example).

### 2.2. Proposed Experimental Protocol

#### 2.2.1. Sample Preparation

**Figure 4.**
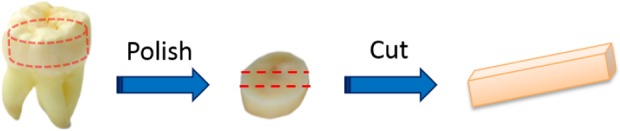
Scheme of sample preparation.

A dentin specimen was obtained from a sound human third molar. The specimen was polished progressively by 80, 500, 800, 1200, 1400, 2000, 2400, 4000 grit SiC polishing papers under water irrigation, and finally turned to cloth polishing with 1 *μ*m diamond suspension liquid for both sides. Then, the dentin slice was cut into one beam with a line cutting saw (Isomet low speed saw, BUEHLER Co., Lake Bluff, IL, USA) (Figure 4). After cutting, the new surfaces were then polished with the same previous polishing steps. Finally, the beam dimensions were controlled as 8 mm *×* 1.38 mm *×* 1.15 mm. Before each step, the sample was washed in an ultrasonic cleaner for 3–5 min. Then, the specimen was stored in the distilled water below 4 °C until the test.

#### 2.2.2. Three-Point Bending Setup

**Figure 5.**
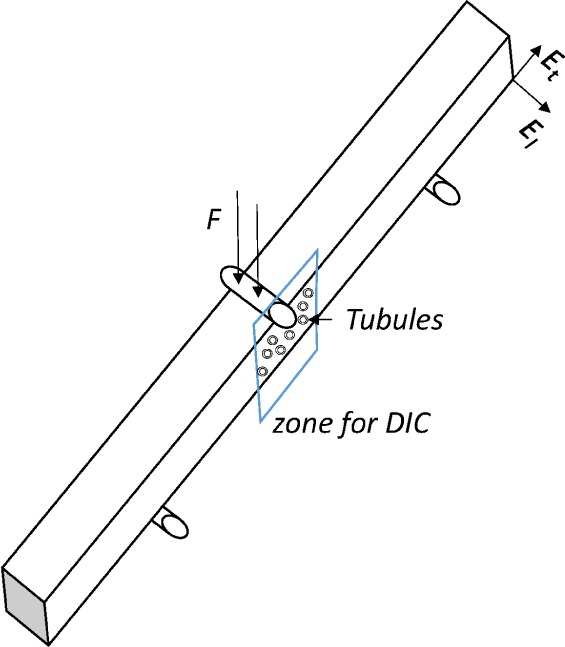
Three-point bending test under the optical microscope.

Three-point bending tests were performed using a bending test device equipped with a 1 kN load cell (MICROTEST, Deban UK, Suffolk, UK) with a supporting span of 5 mm. Motor speed was selected as 0.2 mm/min. All the setup was mounted under an optical microscope (InfiniteFocus, Alicona Co., Raaba, Austria) in order to get the strain distribution of the beam side surface located below the force-indenter as shown in Figure 5. The direction of the load is applied perpendicular to the tubular orientation.

#### 2.2.3. DIC

The software *correli_q4* [21] was used to analyze the strain distribution comparing the reference and deformed images. The deformed image was taken under the load force of 60 N. The element size for DIC was selected as 64 pix.

#### 2.2.4. Determination of the Elastic Modulus and *σ_max_*

The mechanical setup is placed under an optical microscope in order to get images of a selected area of the sample (Figure 5) at different loads. Strain estimates are obtained by DIC and the stresses are estimated by FEA. The edge load can be known from the three-point bending test device. With the maximum total force, one can obtain the maximum tensile stress by FEA. During the bending test process, the strain is evaluated by DIC. Using stress and strain estimates in direction *x* as shown in Figure 1, when the terms *σ_yy_* and *σ_zz_* are much lower than *σ_xx_*, the elastic modulus in direction *x* (*E_t_*) can be derived by Hooke’s law:

$$E_{t}=\frac{\sigma_{xx}}{\varepsilon_{xx}}$$

In our case, the maximum total force found in the experiment (*F_max_* = 65 *N*) was used to determine the maximum bending stress. Tensile test and bending test are known to give slightly different results even when the material fails in tension in both tests, which is why the bending test results in term of strength are called flexural or bending strength and not tensile strength. In this article, we will estimate the maximum tensile stress in the experiment and call it “flexural strength”, as is usually done.

**Figure 6.**
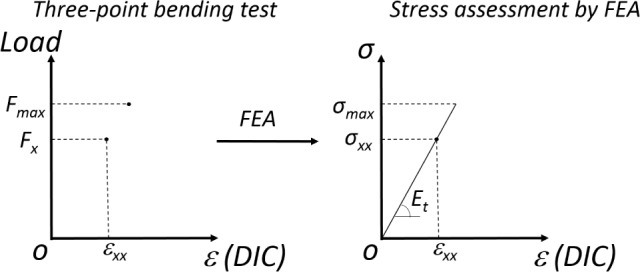
Method of the determination of elastic modulus and of the maximum stress values *σ_max_*.

A total force of 60 N (*F_x_*) was used to determine the stress in order to obtain the elastic modulus, as shown in Figure 6.

## 3. Results and Discussion

### 3.1. Recalculation of the Stresses in Bending Test Configurations Found in the Two Selected Articles

#### 3.1.1. Three-Point Bending Test [13]

**Figure 7.**
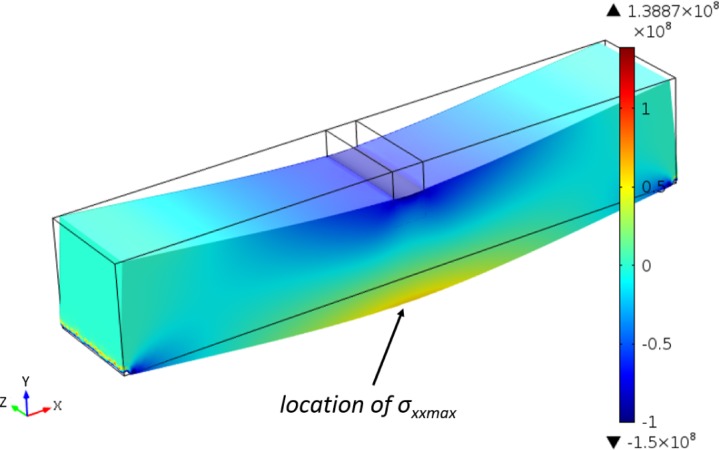
The *σ_xx_* distribution in the beam (unit for color scale bar: Pa).

With the beam dimensions in three-point bending test of Rees *et al.* [13], using the Bernoulli–Euler equation, the maximum tensile stress (often called maximum bending or flexural stress) with assuming boundary force (*F* = 50 *N*) is calculated as 103.1 MPa, whereas, using FEA, the maximum tensile stress is 58.2 MPa. Figure 7 shows the distribution of *σ_xx_* in three-point bending test. As expected, there is a neutral plan in the middle of the beam, and the values are antisymmetric relative to the stress direction (compression and tension). The maximum tensile stress is located at the outside surface of the beam.

#### 3.1.2. Four-Point Bending Test

**Figure 8.**
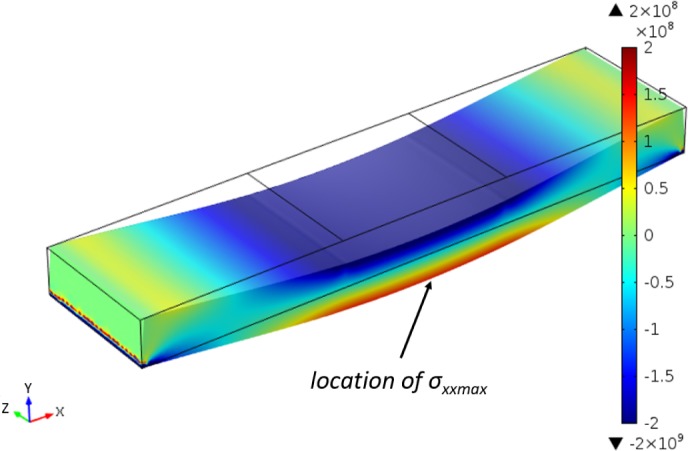
The *σ_xx_* distribution map of four-point bending test (unit of color scale bar: Pa).

According to the Bernoulli–Euler beam equation, the maximum tensile stress for crown and root dentin samples (loads of 5 and 12 N, which are the maximum loads found by Eltit *et al.* [19]) were 136.7 and 328.1 MPa, which matched the values expressed in [19]. However, by using FEA, the maximum tensile stress for coronal and root dentin test is found as 59.1 and 141.8 MPa, respectively. The *σ_xx_* distribution map was shown in Figure 8 with an edge load of 12 N. The maximum bending stress can be found in the outside edge of the beam withstanding tensile stress.

### 3.2. Experimental Results

**Figure 9.**
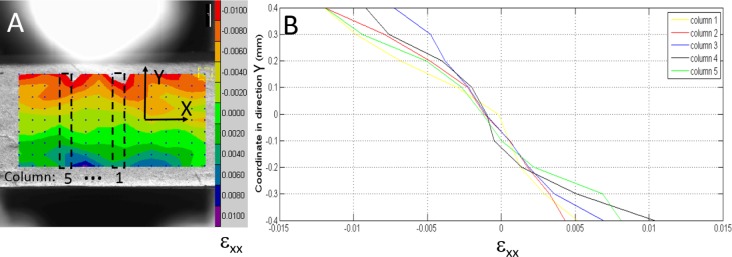
DIC strain distribution for intact dentin beam. (**A**) Three-point bending test and selected areas to acquire *ε_xx_* using DIC; (**B**) *ε_xx_* distribution along direction *y* (from DIC).

The strain distribution map along direction *y* obtained by DIC is shown in Figure 9. Note that *ε_xx_* shows a linear relationship with respect to the local coordinate in direction *y*. Five areas (columns 1 to 5) were selected to evaluate *ε_xx_* distribution, which is approximately antisymmetric relative to the middle neutral plan as expected. In the same positions, stress distributions were assessed by FEA. Thus, the mean elastic modulus was found as 11.9 GPa.

**Figure 10.**
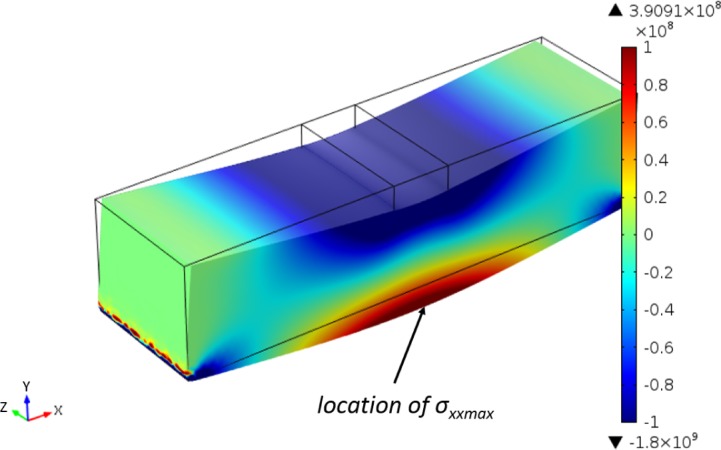
The *σ_xx_* distribution estimation (unit of color scale bar: Pa).

With the maximum boundary load of 65 N found in the experiment, the stress distribution map is obtained by FEA with beam dimensions corresponding to our dentin sample. Tensile stresses are logically located under the neutral plan. On the location marked in Figure 10, *σ_xx_* was found as 143.9 MPa, and at this location *σ_yy_* and *σ_zz_* were found to be below 0.4 MPa, which were much lower than *σ_xx_*, hence the possibility to apply the formula $E_{t}=\frac{\sigma_{xx}}{\varepsilon_{xx}}$.

**Figure 11.**
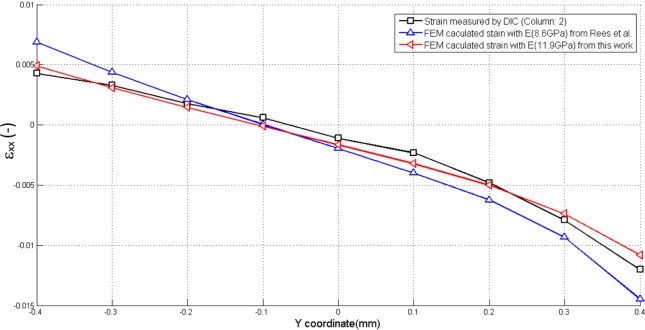
The comparison of *ε_xx_* between FEM and DIC along the *y* coordinate.

In order to validate our method, the deformations measured by DIC and FEM (using our experimental result and the result from Rees *et al.* (1994) [13]) were compared (Figure 11). Strain distribution along *y* coordinates under the indenter (column 2 in Figure 9) measured by DIC was selected as an example.

### 3.3. Discussion

Large discrepancies have been found in the literature concerning dentin mechanical properties (and especially its Young’s modulus). It is probably caused by stress and strain assessment errors. Indeed Bonfield and Datta [22] compared the value of elastic modulus for compact bone with two kinds of micro-strain measuring techniques: they have shown that with different strain measurements, the value of elastic modulus clearly varied. It is likely to be the same while assessing dentin mechanical properties. The elastic modulus is also very sensitive to the strain rate. As reported by Jantarat *et al.* [12] and McElhaney [23], great differences of elastic modulus can be observed due to the strain rate variation (from 0.001 to 1500 per second).

From the recalculation of selected articles, it can be inferred that the Bernoulli–Euler beam equation is probably used to get the flexural strength and elastic modulus. Since the Bernoulli–Euler beam theory is based on the hypothesis of a large slenderness ratio, it can lead to an inaccurate assessment of stresses when the slenderness ratio is inadequate. This would also lead to an error of evaluation of the modulus. Compared with the results of four-point bending test of Eltit *et al.* [19], the maximum bending stress of root dentin obtained from classical beam equation was reported at more than 300 MPa. This is in contradiction with the maximum tensile stress deduced from uniaxial tension test [5,6,8]. However, the maximum bending stress of 55 and 132 MPa (for root and coronal dentin, respectively) deduced using FEA stress assessment seems to better match the results from the literature value, which reported a value of 31 to 130 MPa under uniaxial tension tests [1,5,6]. Thus, it can be inferred that with such sample dimensions, it is inaccurate to use the classical beam theory to assess the stress distribution inside the beam, and that would also lead to an incorrect estimate of the elastic modulus. Nevertheless, elastic modulus determined with beam theory can be used as a comparative tool.

For stress determination, the geometry and positioning of sample are crucial. Some non-parallel alignment of the load can occur due to poorly controlled geometry of the sample in uniaxial compression. In order to well bond the sample with the grips of the machine, an adhesive glue can be used in the tension test [5], at the cost of increasing the relaxation effects. Those effects are not taken into account when using the Bernoulli–Euler beam equation to assess stresses but FEA can also well avoid the influence of those effects.

For strain determination, Sano *et al.* [5] and Rees *et al.* [13] used the load-displacement curve from the testing device to get the strain and elastic modulus. Lower values of elastic modulus were reported, which were probably caused by the inaccurate evaluations of strain: the measured displacement includes not only the whole sample displacement but also motions of the loading setup. This leads to the impossibility of comparing the results in different studies done on different machines. In our study, DIC was used to assess the strain on the external surface of the beam for three-point bending. It allows a more global assessment of strain (on a whole surface) with a satisfactory resolution. The accuracy of the DIC technique is determined by the optical resolution of the focusing system used for the image capture. The precision, therefore, mainly depends on the pixel resolution of the camera. The camera in the microscope used in our test has a size of 1280 (horizontal) by 1024 (vertical) pixels, which is higher than in the study of Palamara *et al.* [8], and thus may lead to a higher resolution on the strain measurement. Moreover, according to Figure 11, we can observe that the values from our finite element model match well with our experimental results.

As discussed above, stress and strain assessment is crucial to get accurate estimates of dentin mechanical properties. For three-point bending test, combined FEA for stress estimate and DIC for strain determination may be a method to explore the dentin elastic modulus and maximum tensile stress. Elastic modulus determined by our method is *E_t_* (because the tensile stress direction is perpendicular to the tubular orientation); our test allowed us to measure *E_t_* as 11.9 GPa, and the maximum stress as 143.9 MPa. The elastic modulus measured here matches the results in the references [5,6,8], in which elastic moduli were reported between 6 and 19 GPa (determined by tension tests). Similarly, our maximum tensile stress corresponds to the magnitude of uniaxial tension test results [1,5,6].

However, for dentin sample, the location in the tooth from where the sample comes may significantly influence the properties of the mechanical test. The density of the mineral phase indeed varies due to the change in tubule density and diameter in the different dentin locations. The statistical structure characteristics have been summarized by Marshall *et al.* [1]. The percentage of peritubular dentin in crown dentin varies from 60% near the pulp cavity to 3% at the dentin–enamel junction (DEJ), and the percentage tubule area and diameter vary from about 22% and 2.5 *μ*m near the pulp to 1% and 0.8 *μ*m at the DEJ. The intertubular matrix area varies from 12% at the pulp cavity up to 96% near the DEJ. It can be inferred that there is a sharp variation in the density and diameter size of tubules and of peritubular dentin in the different locations from the pulp to the DEJ [24]. Hence, the variations in the properties of the dentin sample may be explained probably by the mineral phase density changes within the tooth. Tests on bone specimen have found that the elastic modulus is proportional related to the mineral density [25]. As local mineral densities are different in the parts of peritubular and intertubular dentin, indentation tests on dentin samples have shown that the elastic modulus strongly depends on the location [17]. Thus, the bending test measured here can be done on different samples taken from different depths to assess the variation of *E_t_* with depth (and also with mineral density).

To model the dependency of the dentin modulus with its components ratio, a classical composite model can be used. To do this, the local moduli are needed. With an indentation system, Kinney *et al.* [26] have reported significantly different elastic modulus for peritubular (29.8 GPa) and intertubular (18–21 GPa). According to the composite modulus model, elastic modulus in direction *x* can be described as:

$$E_{t}=\frac{E_{p}E_{i}}{E_{p}r_{i}+E_{i}r_{p}}$$

where *E_p_* and *E_i_* are the elastic modulus of peritubular and intertubular dentin, respectively, and *r_i_* and *r_p_* are the area ratios of intertubular and peritubular dentin respectively. Without considering the percentage of tubular area, an approximation of the elastic modulus in direction *x* can be inferred between 18 and 25 GPa. However, the density of the tubules changes a lot within the tooth. With the addition of the tubular ratio in the composite model, the elastic modulus becomes:

$$E_{t}=\frac{1}{\frac{r_{p}}{E_{p}}+\frac{r_{i}}{E_{i}}+\frac{r_{tubule}}{E_{tubule}}}$$

where *r_tubule_* and *E_tubule_* are ratio and modulus for the tubule respectively. Since a very low modulus is expected for the tubules, we can expect a significant decrease of *E_t_*.

Besides, lower values of elastic modulus on dentin have also been found by Marshall *et al.* [1]. The elastic moduli should be more reasonably represented as a range of values rather than one absolute value, or more accurate values should be obtained to show the relation between the exact location within the tooth (and also mineral density).

It is to be noticed that other factors also influence dentinal mechanical properties, such as storage conditions, tooth age and disease. This is why it is crucial to try to standardize the mechanical tests on dentin in order to be able to compare results of different research groups on different dentin samples (storage, age, disease) and make better evaluations.

## 4. Conclusions

Errors in stress and strain assessment during mechanical tests can possibly lead to a misevaluation of the mechanical properties. In this paper, a review of dentinal mechanical properties has been done and large discrepancies were found in the literature.

Two articles of the literature using bending tests were selected and re-examined using FEA to highlight the importance of stress and strain evaluation to get mechanical properties. Then, a new method (using FEA and DIC) is proposed to better evaluate stress and strain distributions, and consequently elastic modulus. To illustrate and prove the feasibility of the method, the elastic modulus and maximum flexural stress were obtained for a dentinal sample, which were respectively 11.9 GPa and 143.9 MPa. The next step of this study is to improve the stress field assessment in the beam by considering dentinal anisotropy in FEA.

Using accurate and robust methods to assess stress and strain is needed to avoid setup-dependent measurements. Keeping this idea as the ultimate objective, an experimental protocol based on a three-point bending experiment under an optical microscope is proposed, which should perhaps trigger discussion on the standardization of dentinal sample mechanical testing. Being able to compare results of different research groups will indeed lead the community to a better understanding of the differences arising from other relevant factors such as species, age, disease, storage condition, *etc.* (instead of comparing indirectly their equipment).

## Figures and Tables

**Table 1.** Literature review of the mechanical properties of human dentin (elastic modulus units: GPa, stress unit: MPa).

Methods	*E_l_*	*E_t_*	*σ_bending_*	*σ_compression_*	*σ_tension_*	*ε* measure	*σ* measure
RUS [3]	25.0	23.3					
RUS [4]	36.0	29.0					
Micro-pillar compression [16]	13.0	3.5		60–160		$\varepsilon =\frac{d}{L}$	$\sigma =\frac{F}{S}$
Micro-pillar compression [17]	16.2	13.2		186–210		$\varepsilon =\frac{d}{L}$	$\sigma =\frac{F}{S}$
Diametrical compression [8]	6.5 ± 2.0	6.5 ± 2.0			50.9–58.7	DIC	$\sigma_{compression}=\frac{6F}{\pi Dt}$
Compression [7,8,10]	10.7 ± 2.4	11.9 ± 3.0		294–333 [8] 230–300 [7,10]		DIC [8] Strain gauge [7,10]	$\sigma =\frac{F}{S}$
Three-point bending [13]		8.7 ± 0.86				Crosshead (deflection assessment)	Beam equation
Four-point bending [19]			145–326			Crosshead (deflection assessment)	Beam equation
Tension [1,5,6]		6.0–19.3			30–130	Strain gauge	$\sigma =\frac{F}{S}$

*d* is the crosshead displacement; *L* is the length of sample in compression direction; *F* is the load from the compression device; *S* is the cross section area of the specimen; *D* is the diameter of cylindrical sample; *t* is the thickness of cylindrical sample.

## References

[1] Marshall G.W., Marshall S.J., Kinney J.H., Balooch M. (1997). The dentin substrate: Structure and properties related to bonding. J. Dent.

[2] Kinney J.H., Marshall S.J., Marshall G.W. (2003). The mechanical properties of human dentin: A critical review and re-evaluation of the dental literature. Crit. Rev. Oral Biol. Med.

[3] Kinney J.H., Gladden J.R., Marshall G.W., Marshall S.J., So J.H., Maynard J.D. (2004). Resonant ultrasound spectroscopy measurements of the elastic constants of human dentin. J. Biomech.

[4] Lees S., Rollins F. (1972). Anisotropy in hard dental tissues. J. Biomech.

[5] Sano H., Ciucchi B., Matthews W.G., Pashley D.H. (1994). Tensile properties of mineralized and demineralized human and bovine dentin. J. Dent. Res.

[6] Bowen R.L., Rodriguez M.S. (1962). Tensile strength and modulus of elasticity of tooth structure and several restorative materials. J. Am. Dent. Assoc.

[7] Peyton F., Mahler D., Hershenov B. (1952). Elastic and mechanical properties of human dentin. J. Dent. Res.

[8] Palamara J.E.A., Wilson P.R., Thomas C.D.L., Messer H.H. (2000). A new imaging technique for measuring the surface strains applied to dentine. J. Dent.

[9] Stanford J.W., Weigel K.V., Paffenbarger G.C., Sweeney W.T. (1960). Compressive properties of hard tooth tissues and some restorative materials. J. Am. Dent. Assoc.

[10] Craig R., Peyton F. (1958). Elastic and mechanical properties of human dentin. J. Dent. Res.

[11] Zaytsev D., Ivashov A.S., Mandra J.V., Panfilov P. (2014). Deformation behavior of human enamel and dentin-enamel junction under compression. Mater. Sci. Eng. C.

[12] Jantarat J., Palamara J.E., Lindner C., Messer H.H. (2002). Time-dependent properties of human root dentin. Dent. Mater.

[13] Rees J., Jacobsen P., Hickman J. (1994). The elastic modulus of dentine determined by static and dynamic methods. Clin. Mater.

[14] Ryou H., Amin N., Ross A., Eidelman N., Wang D.H., Romberg E., Arola D. (2011). Young’s modulus of peritubular and intertubular human dentin by nano-indentation tests. J. Mater. Sci. Mater. Med.

[15] Han C.F., Wu B.H., Chung C.J., Chuang S.F., Li W.L., Lin J.F. (2012). Stress-strain analysis for evaluating the effect of the orientation of dentin tubules on their mechanical properties and deformation behavior. J. Mech. Behav. Biomed.

[16] Ziskind D., Fleischer S., Zhang K., Cohen S.R., Wagner H.D. (2010). A novel experimental method for the local mechanical testing of human coronal dentin. Dent. Mater.

[17] Ziskind D., Hasday M., Cohen S.R., Wagner H.D. (2011). Young’s modulus of peritubular and intertubular human dentin by nano-indentation tests. J. Struct. Biol.

[18] Grimal Q., Raum K., Gerisch A., Laugier P. (2011). A determination of the minimum sizes of representative volume elements for the prediction of cortical bone elastic properties. Biomech. Model. Mechanobiol.

[19] Eltit F., Ebacher V., Wang R. (2013). Inelastic deformation and microcracking process in human dentin. J. Struct. Biol.

[20] Zhang D., Arola D.D. (2004). Applications of digital image correlation to biological tissues. J. Biomed. Opt.

[21] Hild F., Roux S. (2008). CorreliQ4: A software for finite element displacement field measurements by digital image correlation. Rapp. Interne LMT Cachan.

[22] Bonfield W., Datta P.K. (1974). Young’s modulus of compact bone. J. Biomech.

[23] McElhaney J.H. (1966). Dynamic response of bone and muscle tissue. J. Appl. Physiol.

[24] Garberoglio R., Brännström M. (1976). Scanning electron microscopic investigation of human dentinal tubules. Arch. Oral Biol.

[25] Carter D., Hayes W. (1977). The compressive behavior of bone as a two-phase porous structure. J. Bone Jt. Surg.

[26] Kinney J.H., Balooch M., Marshall S.J., Marshall G.W., Weihs T.P. (1996). Hardness and Young’s modulus of human peritubular and intertubular dentine. Arch. Oral Biol.

